# Quantitative Gadolinium-Free Cardiac Fibrosis Imaging in End Stage Renal Disease Patients Reveals A Longitudinal Correlation with Structural and Functional Decline

**DOI:** 10.1038/s41598-018-35394-4

**Published:** 2018-11-19

**Authors:** Tori A. Stromp, Tyler J. Spear, Rebecca M. Holtkamp, Kristin N. Andres, Joshua C. Kaine, Wissam H. Alghuraibawi, Steve W. Leung, Brandon K. Fornwalt, Moriel H. Vandsburger

**Affiliations:** 1 Department of Physiology, University of Kentucky, Lexington, KY USA; 2 Glaxo Smith Kline Research and Development, Philadelphia, PA USA; 3 Saha Cardiovascular Research Center, University of Kentucky, Lexington, KY USA; 4 College of Medicine, University of Kentucky, Lexington, KY USA; 5 Gill Heart and Vascular Institute, University of Kentucky, Lexington, KY USA; 6 Department of Bioengineering, University of California Berkeley, Berkeley, CA USA; 7 Department of Imaging Science and Innovation, Geisinger, Danville, PA USA

## Abstract

Patients with end stage renal disease (ESRD) suffer high mortality from arrhythmias linked to fibrosis, but are contraindicated to late gadolinium enhancement magnetic resonance imaging (MRI). We present a quantitative method for gadolinium-free cardiac fibrosis imaging using magnetization transfer (MT) weighted MRI, and probe correlations with widely used surrogate markers including cardiac structure and contractile function in patients with ESRD. In a sub-group of patients who returned for follow-up imaging after one year, we examine the correlation between changes in fibrosis and ventricular structure/function. Quantification of changes in MT revealed significantly greater fibrotic burden in patients with ESRD compared to a healthy age matched control cohort. Ventricular mechanics, including circumferential strain and diastolic strain rate were unchanged in patients with ESRD. No correlation was observed between fibrotic burden and concomitant measures of either circumferential or longitudinal strains or strain rates. However, among patients who returned for follow up examination a strong correlation existed between initial fibrotic burden and subsequent loss of contractile function. Gadolinium-free myocardial fibrosis imaging in patients with ESRD revealed a complex and longitudinal, not contemporary, association between fibrosis and ventricular contractile function.

## Introduction

Sudden cardiac death and fatal arrhythmias are leading causes of mortality for patients on routine hemodialysis for end stage renal disease (ESRD)^1^. Increasing evidence suggests a link between left ventricular (LV) hypertrophy^2,3^ and/or fibrosis^4–6^ to fatal arrhythmias and heightened mortality rates in patients with ESRD. While cardiac magnetic resonance imaging (CMR) with gadolinium contrast agents is the clinical standard for fibrosis imaging^7–9^, gadolinium agents are contraindicated in patients with ESRD^10^. In place of direct measurement of tissue fibrosis, measurements of both ventricular structure (LV hypertrophy) and mechanical function including reduced global longitudinal strain (GLS) and diastolic strain rate are used in patients with ESRD^11^. Consequently, investigations into molecular mechanisms of cardiac fibrosis, the role of fibrosis in promoting adverse outcomes, and potential longitudinal treatments in ESRD all rely on either repeated invasive biopsies or surrogate measures of fibrosis.

In a prior cardiac magnetic resonance imaging study we demonstrated a gadolinium-free approach to identify cardiac fibrosis based on changes in magnetization transfer (MT) weighting on cine balanced steady state free precession (bSSFP) images^12^. In patients referred for diagnostic imaging with gadolinium, the normalized signal difference between pairs of differentially MT-weighted bSSFP images (ΔS/S_o_) correlated with the severity of fibrosis when assessed using gadolinium based techniques as a standard^12^. In the current study we expand this method for whole heart quantitative fibrosis imaging in patients with ESRD without bone mineral density loss over one year. We present a method by which to measure global fibrotic burden based on changes in the distribution of ΔS/S_o_ values, termed *divergence*. We probe correlations between fibrotic burden and ventricular hypertrophy or cardiac mechanics. Finally, in a sub-group of patients who returned for follow-up imaging after 1 year we demonstrate quantitative changes in fibrotic burden and examine correlations with changes in left ventricular structure and function.

## Results

Patients with ESRD were recruited from within an ongoing study at our institution^13^. Demographics for patients with ESRD (n = 29) and healthy controls (n = 33) are detailed in Table 1. Groups were well matched in age and gender. Patients with ESRD had larger body mass index than controls (p < 0.001). Hemodialysis vintage in patients with ESRD was 4.8 [2.1, 7.7] years. Six patients had prior failed kidney transplants. Clinical features of patients with ESRD are summarized in Table 2. Twenty three participants from the original study were eligible to complete follow-up within the study period of September 2015-April 2017. Participants who did not return had discontinued dialysis following kidney transplant (n = 2), refused for medical or personal reasons (n = 6), were deceased (n = 2), or were lost to follow-up (n = 3). A total of 11 participants (Supplemental Table 1) completed the follow-up exam at 12.2 ± 0.5 months after baseline (range 10.9–12.7 months).

**Table 1:** Participant Characteristics.

Variable	Healthy Controls (n = 33)	ESRD (n = 29)
*Demographics*		
Age (yrs.)	54.0 ± 10.9	53.7 ± 12.8
Male	15 (45)	14 (48)
White/Caucasian	29 (88)	16 (55)
Black/African American	1 (3)	12 (41)
Asian	0	1 (3)
American Indian	1 (3)	0
Hispanic	2 (6)	0
Body Mass Index (kg/m^2^)	24.2 ± 2.3	31.6 ± 6.8^†^
*Cardiac Structure and Function*		
Left Ventricular Mass Index (g/m^2^)	60.4 ± 13.3	94.3 ± 27.8^†^
Mass:Volume Ratio	1.2 [1.0, 1.5]	1.6 [1.3, 2.0]^†^
Septal Thickness (cm)	0.9 ± 0.2	1.3 ± 0.03^†^
End Systolic Volume (mL)	34.5 [25.3, 46.0]	36.5 [31.5,54.4]
End Diastolic Volume (mL)	92.3 ± 25.3	116.5 ± 41.0*
Ejection Fraction (%)	60.5 [56.0, 64.4]	63.8 [58.4, 68.1]
Heart Rate (bpm)	60.8 ± 8.8	72.3 ± 10.8^†^
QRS Duration (ms)	90.0 [84.0, 98.0]	94.0 [77.0, 104.0]
QTc interval (ms)	422.7 ± 18.4	463.0 ± 37.7^†^

Continuous variables presented as mean ± standard deviation or median [interquartile range]. Categorical variables presented as count (%).*p < 0.01, ^†^p < 0.001.

**Table 2:** Clinical Features of Patients with ESRD.

Clinical Features	Results
Dialysis Duration (years)	4.8 ± 3.2
*Primary Etiology of End Stage Renal Disease*	
Diabetes	12 (41)
Hypertension	7 (24)
Glomerulonephritis	2 (7)
Reflux Nephropathy	2 (7)
Obstructive Nephropathy	1 (3)
Interstitial Nephritis	1 (3)
Focal Segmental Glomerulosclerosis	1 (3)
Unknown	3 (10)
*Comorbidities*	
Hypertension	27 (93)
Diabetes	17 (59)
Prior Myocardial Infarction	4 (14)

Continuous variable presented as mean ± standard deviation. Categorical variables presented as count (%).

### Imaging of Cardiac Fibrosis

**Figure 1:**
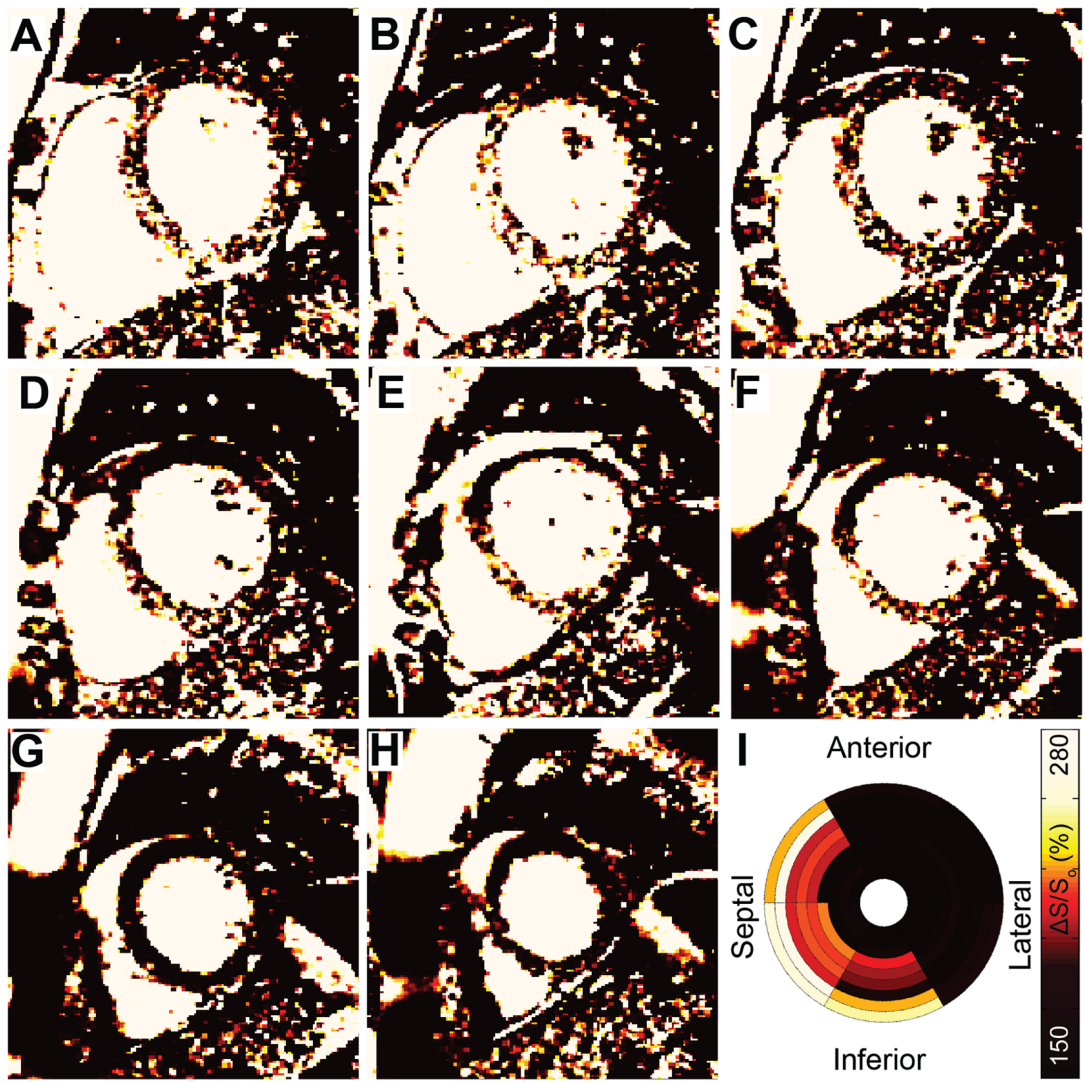
Representative ΔS/S_o_ maps from an ESRD patient. (**A**–**H**) ΔS/So maps from the left ventricular base towards the apex in a patient with ESRD demonstrate a gradient of elevated ΔS/S_o_ across slice positions. Elevated values consistent with dense scar appear in the septal mid-wall at the base of the left ventricle (**A**–**C**). At the mid-ventricle (**D**,**E**) ΔS/S_o_ values are consistent with diffuse fibrosis in the septum and normal tissue in the free wall. Towards the apex (**G**,**H**) ΔS/S_o_ values are consistent with healthy tissue throughout the myocardium. (**I**) The corresponding bulls-eye plot illustrates patterns of elevated mean ΔS/S_o_, particularly in septal segments. Global mean ΔS/S_o_ was 170% and divergence was 37.0 AU.

**Figure 2:**
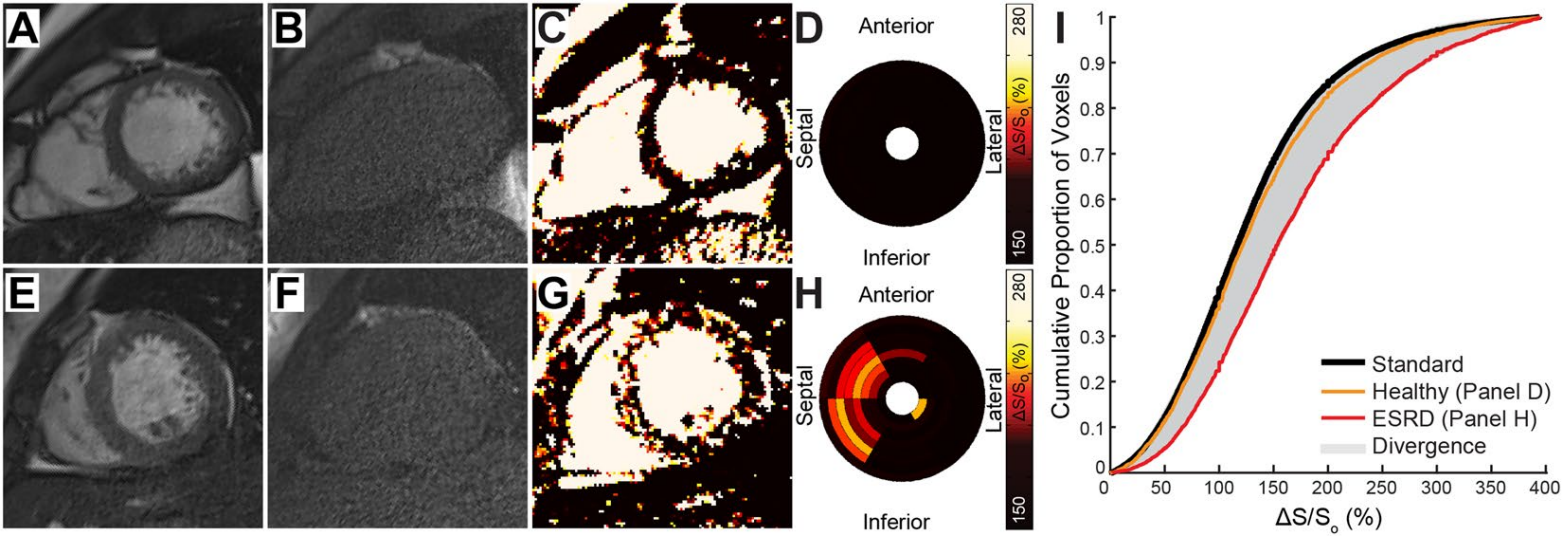
MT-weighted CMR for Tissue Characterization. End diastolic cine bSSFP images acquired with a 45° excitation flip angle in a (**A**) representative healthy control and (**E**) patient with ESRD are paired with images acquired at excitation flip angle of 5° (**B** and **F**, respectively). (**C**,**G**) Maps of ΔS/S_o_ are generated as ΔS/S_o_ = (S_45_ − S_5_)/S_5_ * 100 (%). (**C**) ΔS/S_o_ maps from healthy controls demonstrate low and uniform ΔS/S_o_ values. (**G**) Elevations in ΔS/S_o_ observed in patients with ESRD are consistent with those seen in fibrotic tissue (see^12^). (**D**,**H**) Segmental average ΔS/S_o_ values from LV base (outer ring) to apex (inner ring) are represented using a bullseye plot. Compared to uniformly low ΔS/S_o_ values of a healthy control (**D**), ΔS/S_o_ demonstrates regional heterogeneity in a patient with ESRD, with elevated values primarily in the septum (**H**). (**I**) Using all LV myocardial ΔS/S_o_ values from the control group, a standard cumulative distribution function was generated for this study (black curve). The simulated ΔS/S_o_ distribution was dynamically resized to match the number of voxels per individual LV and compared to the distribution of ΔS/S_o_ values for that individual. Cumulative distributions from the representative healthy control (orange) and ESRD patient (red) are shown. The area between the simulated and participant’s observed distribution represents the global shift in ΔS/S_o_ values and is termed *divergence*. Measurement of divergence integrates the presence of diffuse elevations caused by tissue fibrosis over the entire LV.

Representative MT-weighted CMR maps from a patient with ESRD (Fig. 1) demonstrate diffusely elevated ΔS/S_o_ values, particularly in the septum and towards the base of the left ventricle. The color scheme for presentation of ΔS/S_o_ maps was previously chosen to emulate late gadolinium enhancement, with healthy myocardium appearing dark and densely fibrotic and edematous myocardium appearing bright white^12^. The complete data from a representative healthy control is shown in Supplemental Fig. 1 and demonstrates uniformly normal values throughout the myocardium. Mean global ΔS/S_o_ values were significantly higher in ESRD (145 ± 17%) compared to controls (130 ± 12%, p < 0.001, Fig. S2). We observed all three prevailing patterns of cardiac fibrosis in patients with ESRD including thin scar, diffuse global fibrosis, and focal reactive fibrosis at the ventricular insertion point (Fig. S3). In order to quantify overall changes in fibrotic burden, a standard cumulative distribution of ΔS/S_o_ values was defined by combining all myocardial ΔS/S_o_ values from all healthy participants (Fig. 2). For each individual examined, the standard cumulative distribution was dynamically resized to account for differences in heart size and to match the number of voxels per heart. The individual cumulative distribution function of ΔS/S_o_ values was then compared against the appropriately-sized standard distribution, and the difference between cumulative distributions was integrated across ΔS/S_o_ values as shown in Fig. 2. The resulting value was used as a quantitative marker of fibrosis, termed *divergence* (Fig. 2). A rightward shift is detected when voxels demonstrate increased ΔS/S_o_ values, consistent with increased extracellular volume fraction^12^. Divergence was significantly higher in patients with ESRD (16.3 ± 14.3 AU ESRD vs. 6.5 ± 5.7 AU Control, p = 0.003, Fig. S2), but did not correlate with hemodialysis vintage (ρ = −0.16, p = 0.395).

**Figure 3:**
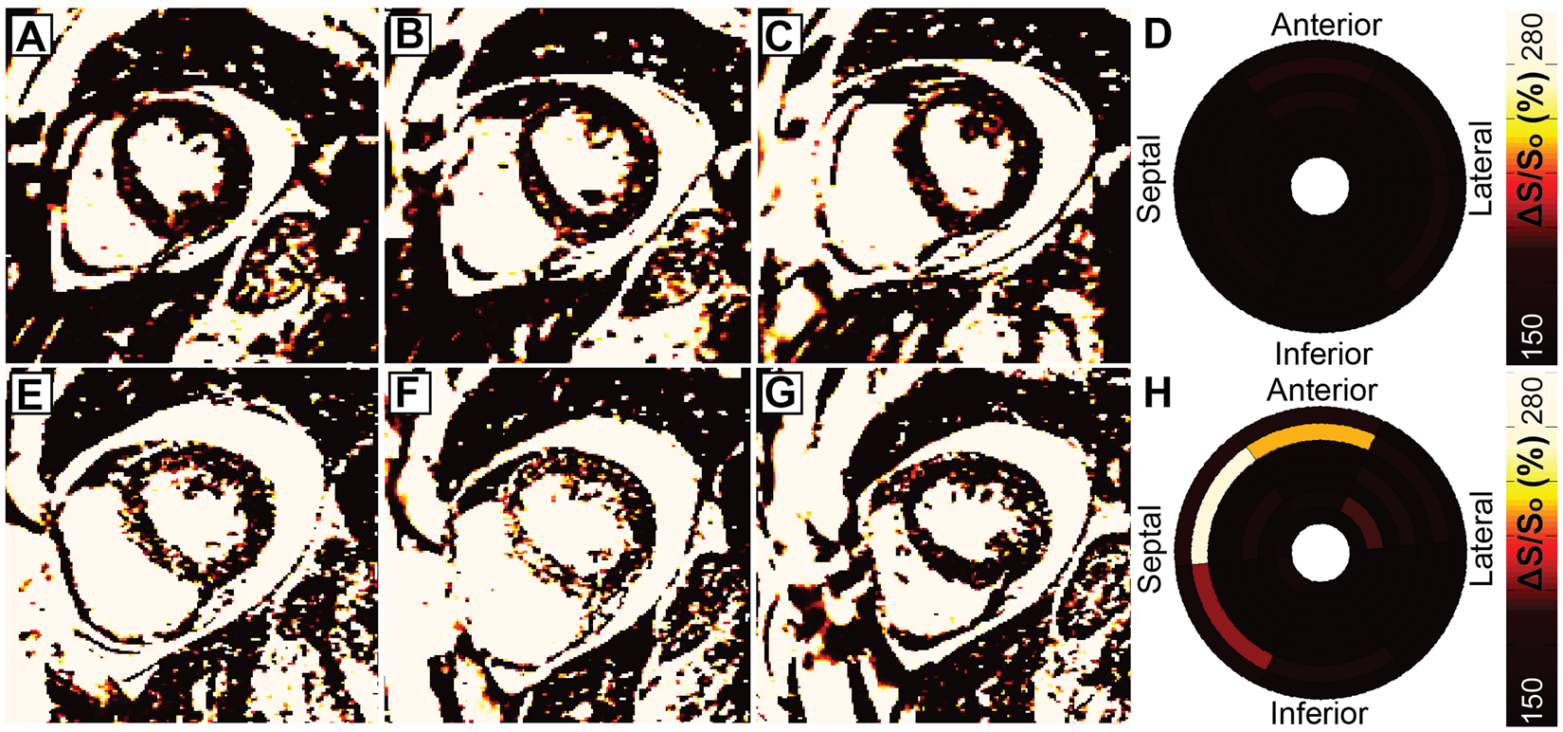
Focal fibrosis development. (**A**–**C**) Images acquired in a 42 year old female patient on hemodialysis for approximately two years with plural effusion demonstrate minor signal elevations but (**D**) low average ΔS/S_o_ across the left ventricle. (**E**–**H**) While global divergence increased by only 8.7 AU at follow-up, regional elevations are evident at the basal and mid ventricular septum. The elevated values are consistent with scar tissue as observed in Stromp *et al*.^12^. Imaging of the same basal/mid-ventricular slice (**B**,**E**) highlights the ability to detect the development of mid-wall enhancement between visits.

Among patients who returned for follow-up imaging, the mean ventricular ΔS/S_o_ values (144.8 ± 19.2% Baseline, 150.0 ± 20.1% Follow Up, p = 0.55) and divergence values (16.9 ± 17.1% Baseline, 20.9 ± 15.3% Follow Up, p = 0.57) did not change substantially from baseline measurements. However, significant heterogeneity was observed between individuals. Specifically, four individuals demonstrated moderate increases in ΔS/S_o_ across a large spatial area that are consistent with increased diffuse fibrosis (Fig. S4), three individuals demonstrated abrupt and substantial increases in regional ΔS/S_o_ values consistent with focal fibrosis (Fig. 3), and 4 individuals showed minimal changes in ΔS/S_o_ values between imaging time points (Fig. S5). A trend of inverse correlation between initial divergence values and subsequent change in divergence over 1 year (ρ = −0.47, p = 0.14) was observed, wherein individuals with initially high divergence values showed little change over one year, and those with low initial divergences demonstrated greater increases over the same period.

### Cardiac Structure, Function, and Mechanics

**Figure 4:**
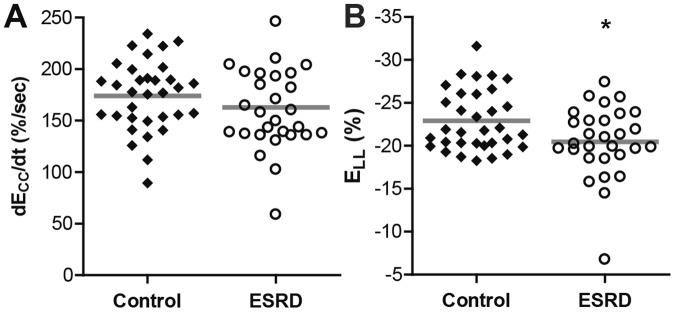
Patients with ESRD demonstrated preserved diastolic circumferential strain rate and reduced global longitudinal strain compared to controls. (**A**) Circumferential diastolic strain rate was similar between ESRD (162.8 ± 41.7%/sec) and control groups (174.1 ± 33.9%/sec, p = 0.247). (**B**) Peak global longitudinal strain (E_LL_) was attenuated in ESRD (−20.5 ± 4.1%) compared to controls (−22.9 ± 3.5%, p = 0.035). Gray bars indicate group means, *p < 0.05.

Ejection fraction was similar between groups (Table 1). Patients with ESRD had increased LVMI and mass to volume ratio (Table 1), with 69% of patients demonstrating values consistent with ventricular hypertrophy. While global circumferential diastolic strain rate was similar between the ESRD and healthy control groups (Fig. 4A), global longitudinal strain was reduced in ESRD (Fig. 4B). Notably, mid-ventricular circumferential diastolic strain rate was unchanged in ESRD (158 ± 51%/s) compared to controls (158 ± 3%/s, p = 0.657). Parameters of cardiac structure and global function (Table S2), chronotropic parameters (Table S2), and both systolic and diastolic strains and strain rates (Table S3) were unchanged after one year among the patients with ESRD who returned for follow-up examination. Only longitudinal diastolic strain rate demonstrated a trend towards decreased magnitude after one year (p = 0.11).

### Cardiac Mechanics and Fibrosis are longitudinally, not concomitantly correlated

**Figure 5:**
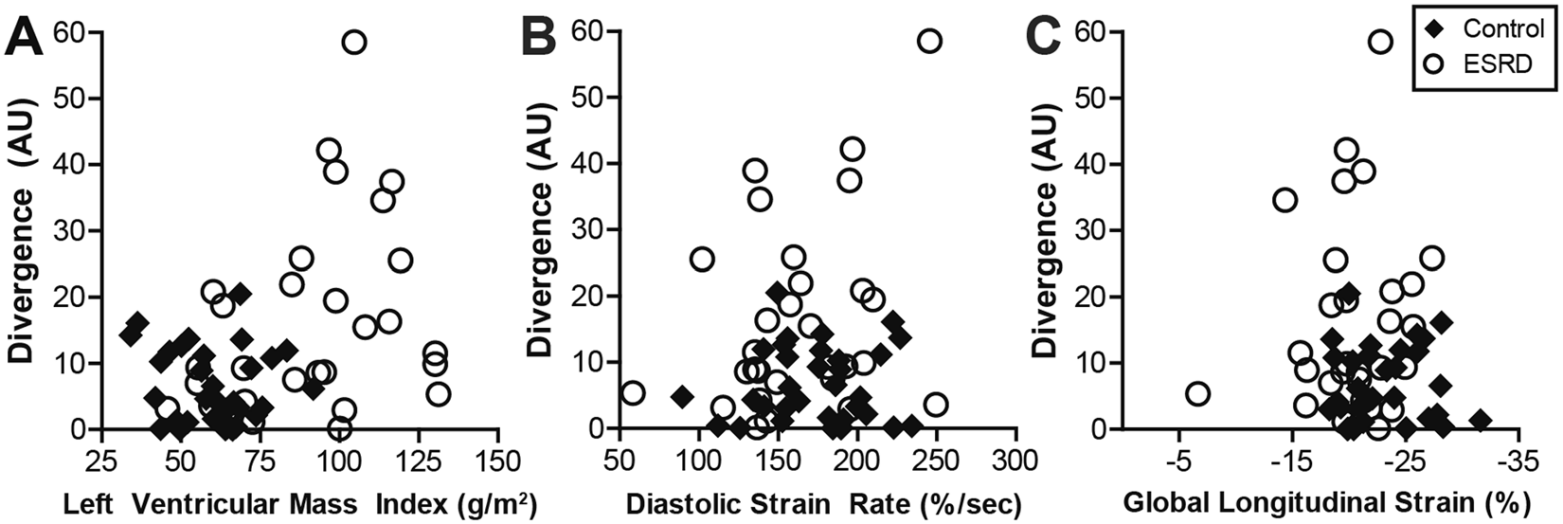
Association of divergence with structural and functional measurements. (**A**) Divergence was moderately correlated with LVMI (ρ = 0.31, p = 0.014), however, among severely hypertrophic individuals (LVMI >100 g/cm^2^) divergence was highly variable. (**B**) Neither diastolic strain rate (ρ = 0.06, p = 0.646) nor (**C**) global longitudinal strain (ρ = 0.02, p = 0.910) correlated with divergence.

**Figure 6:**
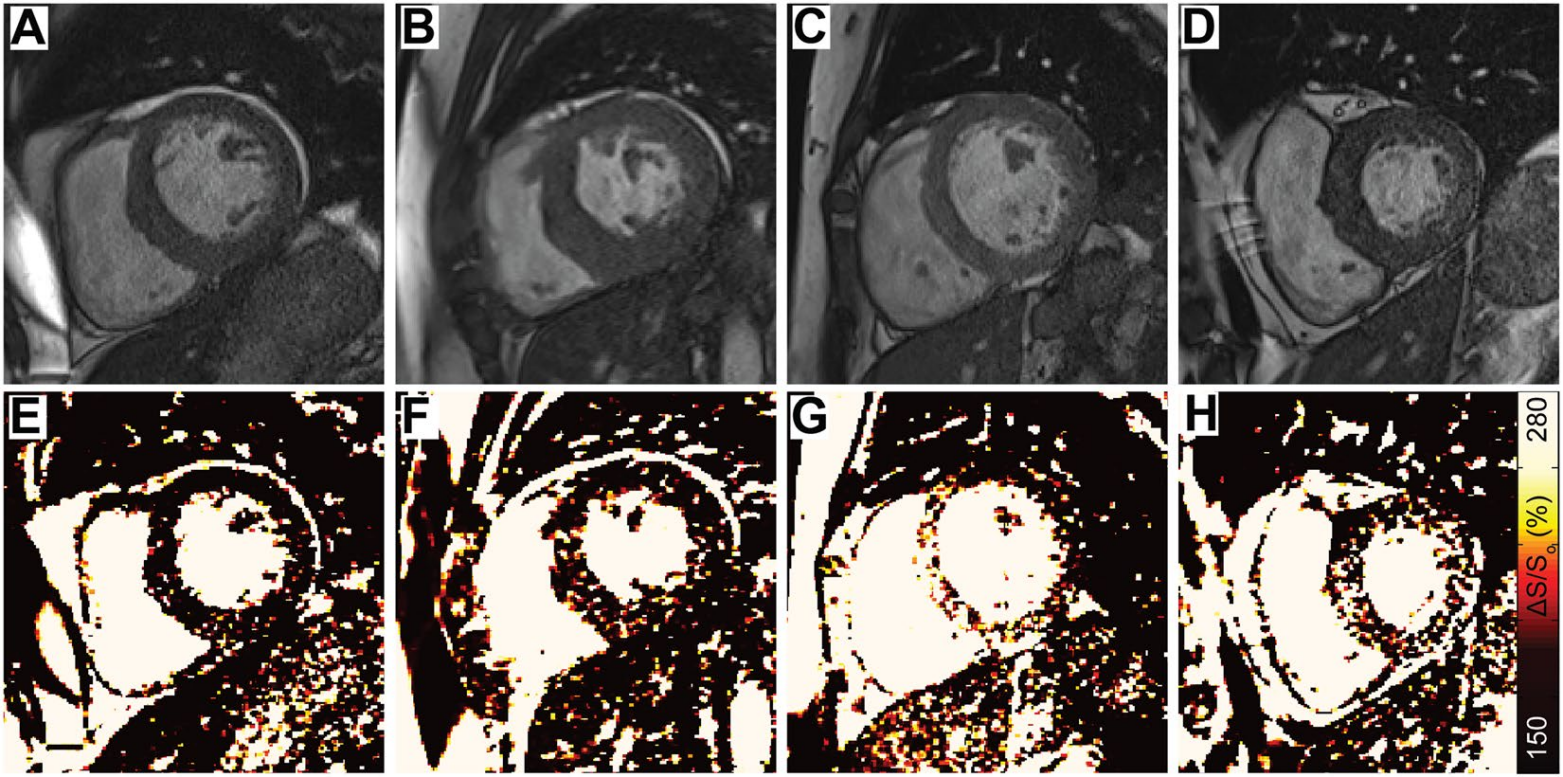
Various spatial patterns of elevated ΔS/S_o_ were observed among hypertrophic patients with ESRD. (**A**–**D**) Representative end diastolic images acquired using a 45° excitation flip angle at the mid-ventricle are shown for 4 hypertrophic patients with ESRD. (**E**–**H**) Corresponding ΔS/S_o_ maps reveal different patterns and magnitudes of ΔS/S_o_ elevation despite the common structural feature of hypertrophy. An expanded version of this figure including 12 patients can be found in the Supplemental Data.

No correlation was observed between divergence and either concomitant diastolic strain rate (ρ = 0.06, p = 0.646) or GLS (ρ = 0.02, p = 0.910, Fig. 5). A moderate correlation was evident when comparing heightened divergence with increased LVMI (ρ = 0.31, p = 0.014, Fig. 5) and septal thickness (ρ = 0.27, p = 0.035). However, many patients with ESRD and significantly elevated LVMI demonstrated normal divergence values (Fig. 5A). This relationship is further illustrated in Fig. 6 by comparison of heterogeneously elevated ΔS/S_o_ patterns in 4 patients with increased septal thickness and heightened LVMI (a larger subset are shown in Fig. S6). In contrast, increased LVMI was significantly correlated with reduced diastolic strain rate (ρ = −0.26, p = 0.043) and attenuated global longitudinal strain (ρ = 0.38, p = 0.002) across participants.

Among patients who returned for follow-up examination, initial divergence values were not correlated with corresponding measures of LVMI (ρ = 0.03, p = 0.92), mass to volume ratio (ρ = 0.16, p = 0.62), global longitudinal strain (ρ = −0.04, p = 0.91), circumferential diastolic strain rate (ρ = 0.37, p = 0.27), or longitudinal diastolic strain rate (ρ = 0.09, p = 0.79). While the correlations between divergence and LVMI (ρ = 0.55, p = 0.08), mass to volume ratio (ρ = 0.18, p = 0.60), global longitudinal strain (ρ = 0.52, p = 0.10), and both circumferential diastolic strain rate (ρ = 0.39, p = 0.23) and longitudinal diastolic strain rate (ρ = 0.42, p = 0.19) were greater after one year, all failed to reach statistical significance in a small sample size.

**Figure 7:**
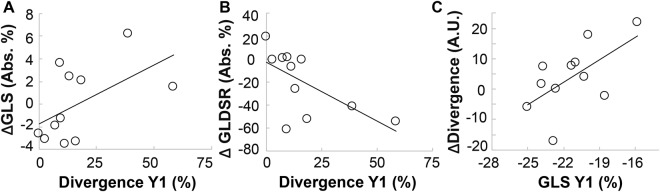
Baseline levels of fibrosis and global longitudinal strain correlated with corresponding parametric changes over one year. Patients with higher baseline divergence values demonstrated greater loss of (**A**) global longitudinal strain (ΔGLS) and (**B**) global longitudinal diastolic strain rate (ΔGLDSR) over one year. (**C**) Interestingly, there was also a correlation between attenuated global longitudinal strain at baseline and subsequent increases cardiac fibrosis over one year. Pearson’s correlation coefficients and statistical values can be found in Table 3.

**Figure 8:**
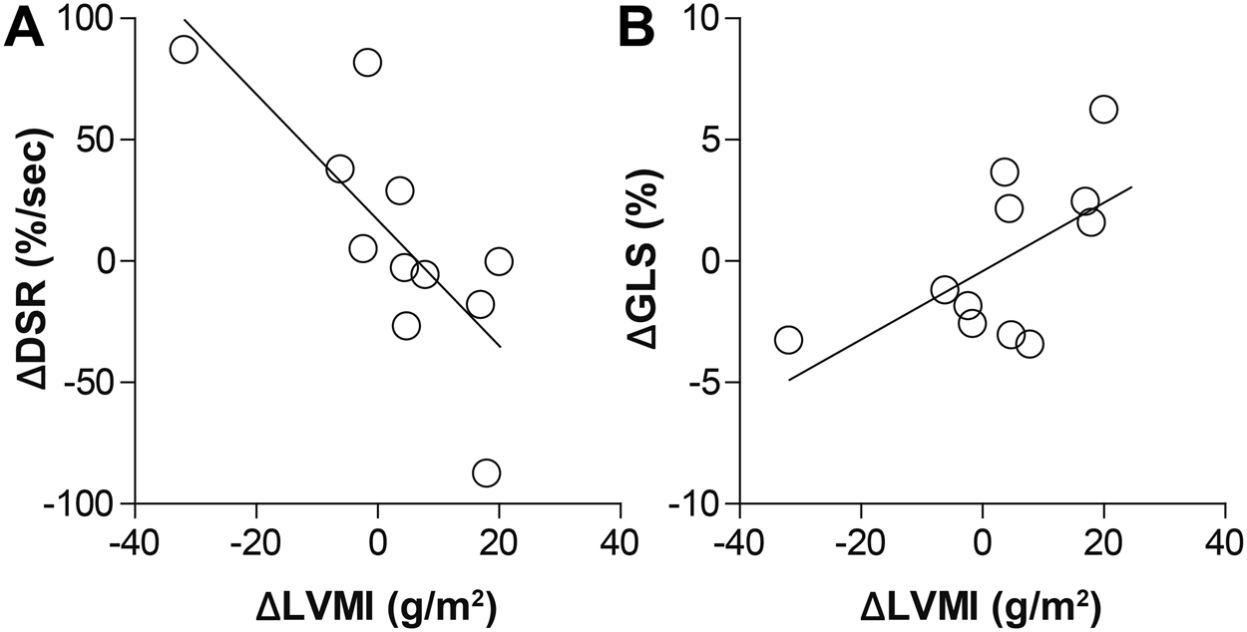
Hypertrophy correlated with attenuation in cardiac mechanics. (**A**) Increases in LVMI over one year strongly correlated with decreases in circumferential diastolic strain rate (r^2^ = 0.58, p = 0.007) and (**B**) with attenuation in global longitudinal strain (r^2^ = 0.38, p = 0.043).

Although divergence and ventricular structure/mechanics were not concomitantly correlated, there was a significant correlation between initial divergence values and corresponding changes in both circumferential and longitudinal diastolic strain rates over one year (Table 3 and Fig. 7). A similar trend was observed with respect to changes in global longitudinal strain, but failed to reach statistical significance (Table 3). Interestingly, when examining changes in divergence between examinations, only the initial magnitude of global longitudinal strain correlated with subsequent changes in divergence (Table 3 and Fig. 7). In addition, individual changes in divergence between visits were not correlated with corresponding changes in LVMI (ρ = 0.43, p = 0.26), GLS (ρ = 0.41, p = 0.21) and circumferential diastolic strain rate (ρ = 0.39, p = 0.24). A strong trend was observed between changes in divergence and corresponding changes in longitudinal diastolic strain rate between examinations (ρ = −0.58, p = 0.0595). However, individual changes in LVMI were significantly correlated with concomitant reductions in GLS (ρ = 0.62, p = 0.043) and circumferential diastolic strain rate (ρ = −0.76, p = 0.007) as shown in Fig. 8. Changes in LVMI and longitudinal diastolic strain rate demonstrated a trend towards correlation (ρ = −0.52, p = 0.099).

**Table 3:** Correlation between initial structure, function, and fibrosis and changes over 1 year.

Year 1	Δ Between Year 1 and Baseline				
	Left Ventricular Mass Index (LVMI)	Diastolic Strain Rate (DSR)	Global Longitudinal Strain (GLS)	Longitudinal Diastolic Strain Rate (LDSR)	Divergence
Divergence	0.45 (0.17)	−0.61 (**0**.**047**)	0.54 (0.089)	−0.63 (**0**.**039**)	−0.47 (0.14)
LVMI	−0.35 (0.29)	0.27 (0.42)	−0.55 (0.083)	0.70 (**0**.**016**)	0.53 (0.091)
MVR	0.42 (0.20)	−0.18 (0.59)	0.22 (0.51)	0.35 (0.29)	0.27 (0.41)
DSR	0.10 (0.77)	−0.50 (0.12)	0.01 (0.99)	−0.28 (0.41)	0.48 (0.13)
GLS	−0.35 (0.29)	−0.17 (0.63)	−0.11 (0.74)	0.11 (0.77)	0.61 (**0**.**046**)
LDSR	0.28 (0.41)	0.13 (0.70)	0.15 (0.65)	0.25 (0.45)	−0.47 (0.15)

Data are presented as Pearson’s ρ and corresponding (p value). P < 0.05 was considered statistically significant. Significant correlations are in bold.

## Discussion

Nearly 40% of mortality among patients with ESRD is attributed to arrhythmias and sudden cardiac death^1^. Prior studies using excised tissue^3,6^ and CMR with gadolinium^4,14^ established cardiac fibrosis as a potential substrate of arrhythmia and sudden death in ESRD^15^. With contraindications to gadolinium in place, ventricular hypertrophy and cardiac mechanics are often used as surrogate imaging markers of cardiac fibrosis, and are also targets for pharmacological reversal in ESRD^16–18^. In this study we used an MT-weighted CMR technique to quantify global ventricular extracellular matrix expansion in patients with ESRD. While we found that patients with ESRD with heightened fibrosis often demonstrate ventricular hypertrophy and attenuated longitudinal and diastolic contractile function, such structural and mechanical measurements were often similarly altered in patients without fibrosis. Additionally, while fibrotic burden was not concomitantly correlated with either ventricular hypertrophy or attenuated mechanical function, greater divergence values at initial examination correlated strongly with subsequent reductions in diastolic strain rates over one year. Further, attenuated initial global longitudinal strain correlated strongly with subsequent increases in fibrotic burden over the same period. Importantly, overall changes in longitudinal diastolic strain rates were highly correlated to changes in LVMI and closely mirrored by changes in fibrotic burden. Together, these findings highlight the complex interplay between global structural and functional changes and local changes in myocardial fibrotic burden in the setting of ESRD.

Late gadolinium enhancement is a standard for identification of myocardial scar and for quantification of scar distribution and burden. Before consensus that patients with ESRD should not undergo late gadolinium enhancement imaging, Schietinger *et al*.^4^ used gadolinium contrast agents to identify three prevailing enhancement patterns in patients with ESRD including thin scar from prior myocardial infarction, diffuse enhancement, and focal fibrosis at a right ventricular insertion point. Similarly, Mark *et al*. observed patterns of thin scar or diffuse enhancement through various regions of the left ventricle in most patients with ESRD^5^. Our previous validation of MT-weighted CMR revealed a strong correlation between elevated ΔS/S_o_ values and late gadolinium enhancement tissue status^12^, and in this study we observed patterns of elevated ΔS/S_o_ similar to the three prevailing patterns described by Schietinger and colleagues (Fig. S3). Among these patterns, diffuse and heterogeneous enhancement of the LV was most common. When regionally diffuse elevations of ΔS/S_o_ occur concurrently with ventricular hypertrophy, calculation of a global mean ΔS/S_o_ value is more likely to obscure potentially meaningful elevations. Given prevailing histological evidence of diffuse cardiac fibrosis in ESRD, we chose to compare cumulative distributions of ΔS/S_o_ for each individual against a standard healthy distribution as a mechanism to integrate small elevations in ΔS/S_o_ that are scattered throughout the left ventricle while simultaneously normalizing for differences in LV mass (Fig. 2). This approach enables quantitation of a spatially diffuse and heterogeneous process and enabled longitudinal comparison of global ventricular fibrotic burden without the need for biopsy.

Increased collagen content in fibrotic myocardium is associated with greater tissue stiffness, cardiomyocyte hypertrophy and reduced LV contractile function^19^. Subsequently, both hypertrophy and mechanical function are often used surrogate imaging markers of fibrosis in studies of patients with ESRD. Ventricular hypertrophy is highly prevalent among patients with ESRD (up to 80%) and associated with poorer prognosis^5,17^. In agreement with prior studies^5,20,21^, we observed substantial hypertrophy in patients with ESRD, yet only a moderate correlation was observed between simultaneous measurements of LVMI and divergence. Similarly, reduced global longitudinal strain in patients with ESRD has been demonstrated using both CMR^22^ and echocardiography^23–25^. While we also observed reduced mean GLS in patients with ESRD compared to healthy controls, most values remained well within the normal variation of healthy cohorts^11,26^. Further, comparing divergence against simultaneous measurements of both GLS and circumferential diastolic strain rate failed to reveal meaningful associations, similar to reports in hypertrophic cardiomyopathy^27^. While ventricular fibrosis and reduced strain have been correlated in individuals without ESRD^28–30^, measurement of contractile function in ESRD is complicated by hemodynamic fluctuations due to timing relative to hemodialysis^23,24^. Such fluctuations have been demonstrated to introduce sampling bias to the measurement of contractile function with echocardiography^31,32^. Further, reduced contractile function has not consistently correlated with either histological measures of fibrosis in the ESRD population^32^, or heart failure or mortality in patients with ESRD^33^. A distinct finding of this study is that the change in fibrotic burden over one year was variable among patients with ESRD but correlated with the degree of attenuation of GLS at the time of initial examination. While this may suggest that reduced contractile function promotes cardiac fibrosis, Hayer and colleagues recently reported that noticeable loss of GLS occurred over one year in 30 early-stage chronic kidney disease patients, despite preserved and normal left ventricular mass and extracellular volume^11^. Further, we observed that loss of contractile function was highly correlated to increases in ventricular mass. Together, these findings highlight the intrinsic difficulty of using discrete measurements to assess whether and how tissue fibrosis, hypertrophy, and loss of contractile function are synergistic or merely concomitant pathological processes in the setting of ESRD.

Several limitations to our study should be noted. First, while MT-weighted imaging of cardiac fibrosis has previously been compared to late gadolinium enhancement, it has not been validated directly against histology in large animals or humans. This should be performed as part of further validation of fibrosis in the setting of kidney disease. Further, this study was performed at 1.5 T where the impact of B1 heterogeneity is modest when compared to 3 T. Extension of this technique to 3 T scanners will likely require alternate MT-weighting methods, or restrictive analysis to the septum. In addition, since the initial goal of our study was to probe for tissue fibrosis in patients with ESRD we used healthy age matched adults as our control population. Second, acquisition of image pairs requires two consecutive breath holds. To mitigate the impact of potential motion, image registration was performed prior to calculation of ΔS/S_o_. Third, age-related extracellular matrix expansion^34^ may have impacted both groups, unrelated to underlying ESRD status. Further, while reflecting proportions within the ESRD population^1^, group differences in race may have led to imbalanced risk factors and confounded measures of cardiac function^2^. An additional major limitation to this study is the small sample of patients who returned for follow up examination after one year. Among those who were eligible for follow up (alive and without kidney transplant) but did not participate in the second scan, many cited deteriorating overall health as a major barrier to participation. Subsequently, a recruitment bias towards healthier patients may have obscured correlations between fibrosis, structure, and function over time that would have been observed in a larger ESRD cohort. It is entirely possible that with a larger cohort we may have seen a strong trend towards universal fibrotic progression among patients with ESRD, or greater concomitant correlations. Further, we did not have the statistical power within this group to examine whether heterogeneity in progression of fibrosis was caused by differences in underlying demographic factors including hemodialysis vintage, comorbidities, or medical history. A larger sample size may have also revealed stronger correlations between initial strain values and subsequent changes. In addition, we did not have access to patient dialysis records and could neither control for, nor examine the potential impact of changes in fluid maintenance between visits.

## Conclusions

We applied MT-weighted CMR and developed quantitative methods for myocardial tissue characterization in patients with ESRD. Comparing divergence with LVMI and global longitudinal strain by CMR confirms that the association between extracellular matrix remodeling, cardiomyocyte hypertrophy, and attenuated contractile function is complex. The ability to non-invasively derive all three measures from CMR can empower the study of heart failure, prognosis, and mortality in the setting of ESRD. Future studies could utilize this combination to examine the acute impacts of dialysis initiation, or to test specific biomarkers of fibrotic progression in the setting of ESRD.

## Methods

### Participants and Recruitment

Patients on routine hemodialysis for ESRD were prospectively referred from the University of Kentucky Healthcare Department of Nephrology if they had no arrhythmia nor CMR incompatible metal or devices (n = 33). Healthy volunteers without cardiovascular disease, hypertension, diabetes, obesity, tobacco use, or CMR incompatibilities were prospectively included if within the age range of our patients (n = 44). Voluntary informed consent was obtained prior to participation. Participants were excluded from data analysis if unable to hold their breath or complete the MRI protocol (n = 1 ESRD each). Body mass index >26 kg/m^2^ in healthy controls (n = 3), abnormal electrocardiogram (n = 1), discovery of aging related (n = 1) or congenital abnormalities (n = 1), or hardware failures discovered after imaging (n = 5) were additional grounds for exclusion. The protocol was approved by the University of Kentucky Institutional Review Board. All research was performed in accordance with relevant guidelines and regulations.

### Cardiac MRI Protocol

A 12-lead electrocardiogram was conducted prior to MRI. Imaging occurred on non-dialysis days with a 1.5 T Siemens Aera scanner (Erlangen, Germany) using an 18-channel body matrix coil and 12-channel spine coil. MT-weighted CMR was completed as previously described^12^. During separate end expiratory breath holds, cine bSSFP image pairs were obtained using excitation flip angles of 5° and 45° in a short axis stack from LV base to apex. Parameters included TE: 1.2 ms, TR: 3.2 ms, bandwidth: 930 Hz, matrix: 256 × 256, in-plane resolution: 1.02 × 1.02 mm, slice thickness: 8 mm, and GRAPPA 2.

### Structure, Function and Strain Analysis

Ventricular volumes and mass were analyzed from short axis images in Argus Viewer (Siemens Healthcare, Erlangen, Germany). Circumferential and longitudinal strains were assessed via custom feature tracking algorithm^35^ (Fig. S7). Global longitudinal strain was measured from a 4-chamber long axis cine image.

### Quantification of ΔS/So Enhancement

Images were analyzed in MATLAB (2014a, The MathWorks Inc., Natick, MA). Cine image pairs with excitation flip angles of 45° (Fig. 1A and E) and 5° (Fig. 1B and F) were registered when necessary to align the LV myocardium. Maps of ΔS/S_o_ (Fig. 1C and G) were generated as described^12^ for each slice using the formula ΔS/S_o_ = (S_45°_ − S_5°_)/S_5°_ * 100 (%), where S is signal intensity per voxel. Figures are presented with a 2 × 3 median filter and color schemes that emulate late gadolinium enhancement images as described previously^12^. The LV myocardium was manually segmented and end diastolic unfiltered ΔS/S_o_ maps were used for quantitative analysis. Bullseye plots demonstrate mean ΔS/S_o_ per sector (Fig. 2D and H). Mean ΔS/S_o_ per individual was calculated from all myocardial voxels.

### Statistical Analysis

Statistics were computed using SPSS Statistics (version 22, IBM Corp, Armonk, NY). Shapiro-Wilk and Levene’s tests assessed normality and homogeneity of variance. Student’s t-tests compared group means for heart rate, end diastolic volume, circumferential diastolic strain rate, and ΔS/S_o_. Mann-Whitney U tests compared group body mass index, LVMI, mass:volume ratio, septal thickness, end systolic volume, ejection fraction, QRS duration, QTc interval, GLS, divergence, and serum biomarkers. Baseline and follow-up results were compared using related-samples Wilcoxon signed rank tests for EDV, ESV, and cardiac output. All other results were compared using analysis of variance (ANOVA) with repeated measures. Change in divergence across scans was compared against measures of structure and function using Pearson correlations. Associations of imaging results with measures of structure, function, and serum biomarkers were measured by Pearson correlations. Data are presented as mean ± standard deviation, median [interquartile range], or count (%) where appropriate. *P* < 0.05 was considered significant.

### Ethics approval

Voluntary informed consent was obtained prior to participation. The protocol was approved by the University of Kentucky Institutional Review Board.

## Electronic supplementary material

Supplemental Data

## Data Availability

All data will be made available from the corresponding author upon reasonable request.
