# Supplementary material for: Quantitative Gadolinium-Free Cardiac Fibrosis Imaging in End Stage Renal Disease Patients Reveals A Longitudinal Correlation with Structural and Functional Decline
Source: Sci Rep. 2018 Nov 19;8:16972. doi: 10.1038/s41598-018-35394-4 (PMC6242893; doi:10.1038/s41598-018-35394-4)
Supplement: Supplementary file 1 — Supplemental Data [file 41598_2018_35394_MOESM1_ESM.docx]

# Quantitative Gadolinium-Free Cardiac Fibrosis Imaging in End Stage Renal Disease Patients Reveals A Longitudinal Correlation with Structural and Functional Decline

**Authors:** Tori A Stromp, PhD^1,2^, Tyler J Spear, BA^3^, Rebecca M Holtkamp, BS^3^, Kristin N Andres, BS^4^, Joshua C Kaine, MD^4,5^, Wissam H Alghuraibawi^6^, Steve W Leung, MD^5^, Brandon K Fornwalt, MD/PhD^7^, Moriel H Vandsburger, PhD*^6^

**Supplemental Material**

Supplemental Table 1. Baseline Demographics of 1 Year Follow-Up Subgroup

| Variable | Result |
| --- | --- |
| Age (yrs) | 51.5 ± 7.2 |
| Male | 6 (55) |
| White | 5 (45) |
| Black/African American | 5 (45) |
| Asian | 1 (9) |
| Hemodialysis vintage (yrs) | 4.6 ± 3.4 |
| Systolic Blood Pressure (mmHg) | 143.0 ± 23.7 |
| Diastolic Blood Pressure (mmHg) | 79.4 ± 11.2 |
| Primary Cause of ESRD |  |
| Hypertension | 3 (27) |
| Diabetes | 5 (45) |
| Obstructive Uropathy | 1 (9) |
| Genetic | 1 (9) |
| Unknown/Unsure | 1 (17) |
| Comorbidities | 7 (64) |
| Hypertension | 10 (91) |
| Diabetes | 4 (66) |
| Numerical variables are presented as mean ± standard deviation. Categorical variables are presented as count (%). | |

**
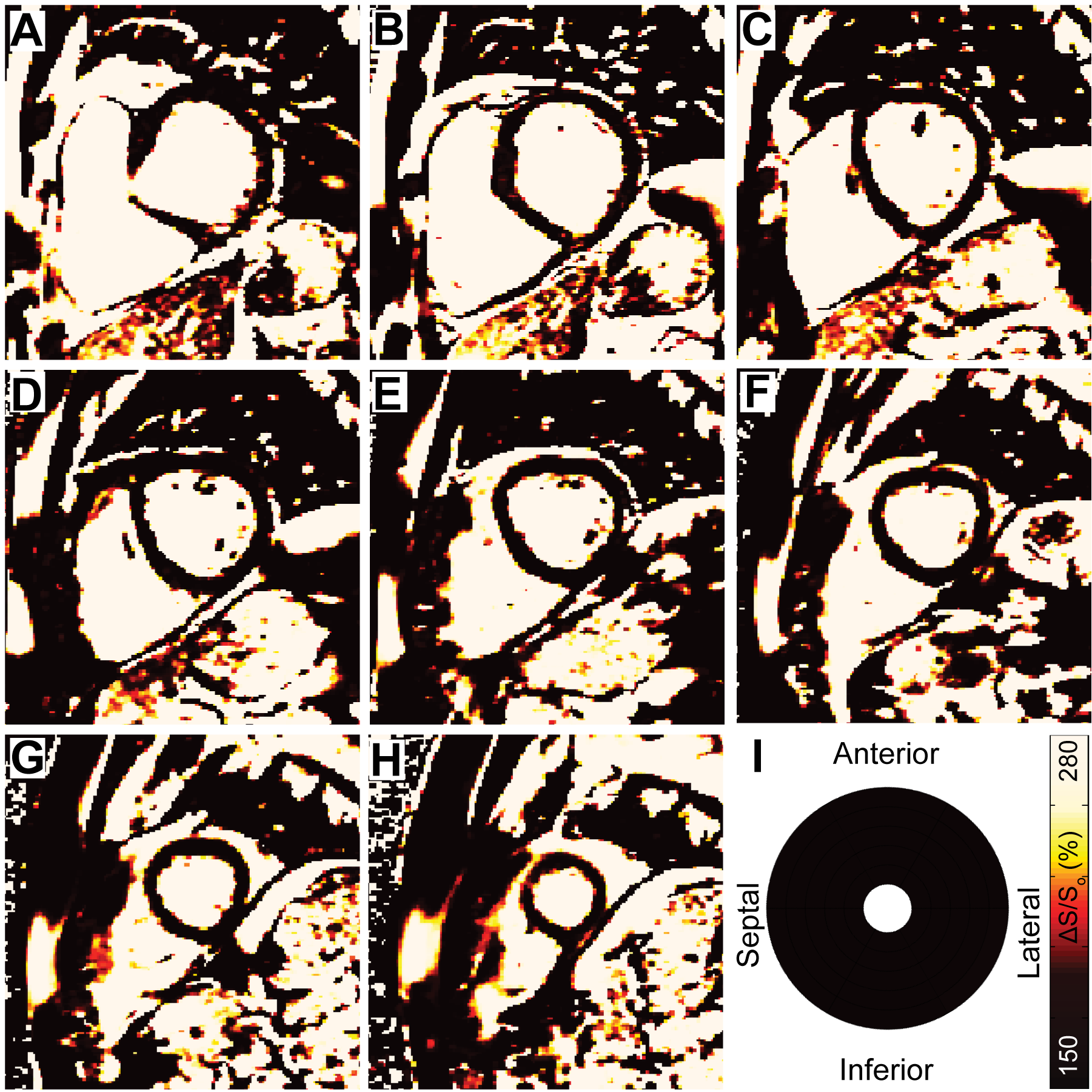
**

**Figure S1. Representative ΔS/S_o_ maps from a healthy participant. (A-C)** Basal, **(D-F)** mid-ventricular, and **(G-H)** apical ΔS/S_o_ maps reveal uniform values consistent with healthy myocardium throughout the ventricle. **(I)** The bullseye plot illustrates low mean ΔS/S_o_ values across all segments of the left ventricle. In this participant, global mean ΔS/S_o_ was 115% and divergence was 4.0 AU.


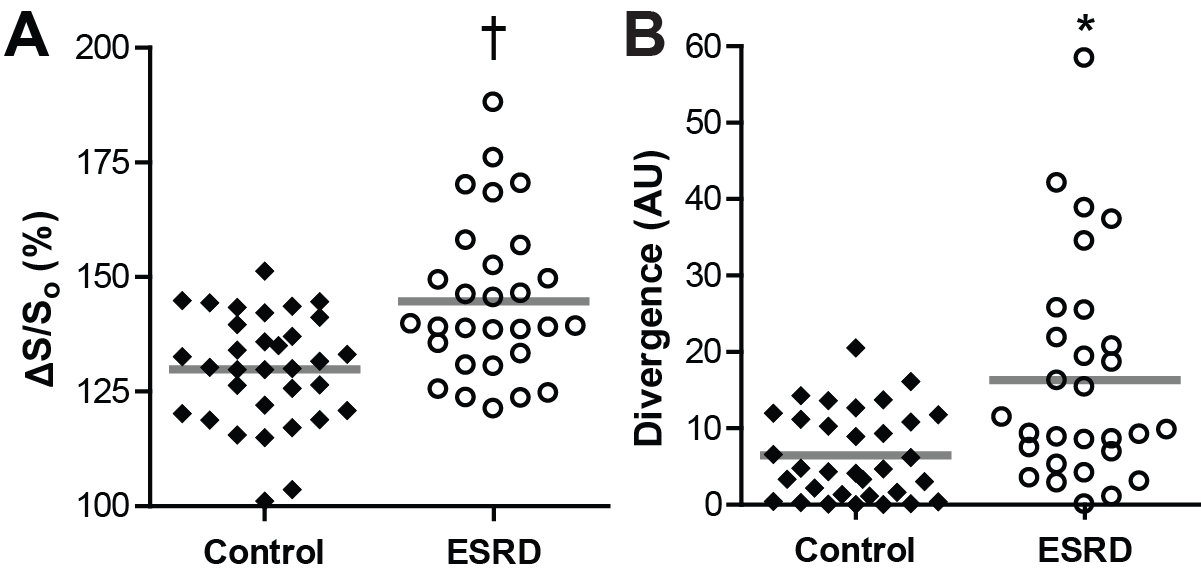


**Figure S2. Quantitative measures derived from MT-weighted CMR were elevated in patients with ESRD. (A)** Average ΔS/S_o_ was elevated in patients with ESRD (144.7 ± 17.1%) compared to controls (129.9 ± 12.0%, p < 0.001). **(B)** Divergence values were increased in patients with ESRD (16.3 ± 14.3 AU) compared to controls (6.5 ± 5.7 AU, p = 0.003). Gray bars denote group means. *p<0.01, ^†^p<0.001.


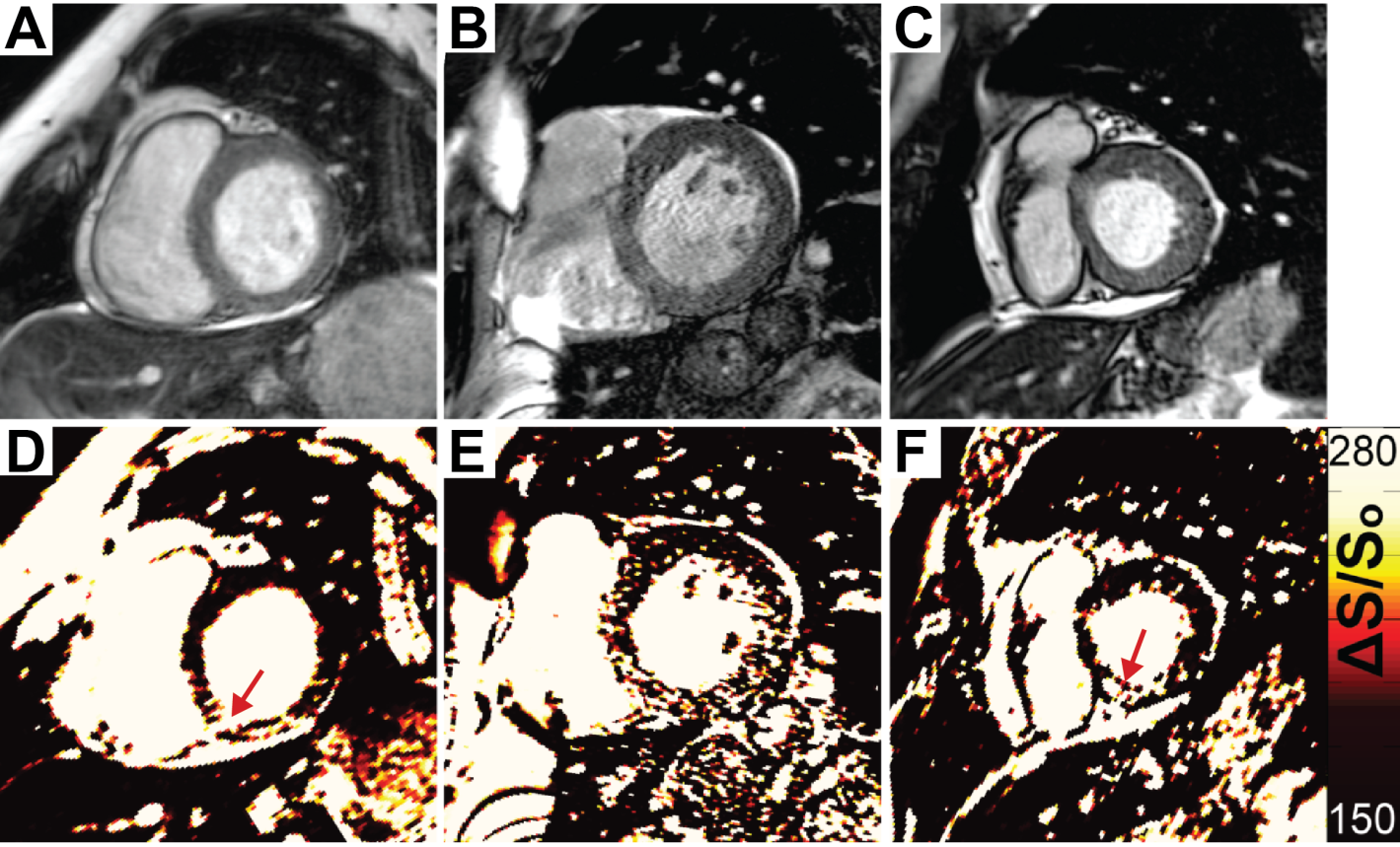


**Figure S3.** **Recapitulation of three prevailing patterns of fibrotic tissue in renal failure patients**.

Prior to the FDA warning and contraindication of gadolinium, Schietinger et al.^4^ observed 3 dominant patterns of late gadolinium enhancement in renal failure patients: thin scar-like enhancement; diffuse fibrosis; and focal fibrosis at the ventricular insertion points unrelated to ischemia. MT-weighted fibrosis imaging in renal failure patients reveals similar patterns to those seen previously using late gadolinium enhancement CMR. **(A-C)** End diastolic bSSFP images in 3 renal failure patients and corresponding maps of ΔS/S_o_ reveal **(D)** thin enhanced tissue, **(E)** diffuse enhancement, and **(F)** focal enhancement at the ventricular insertion point.


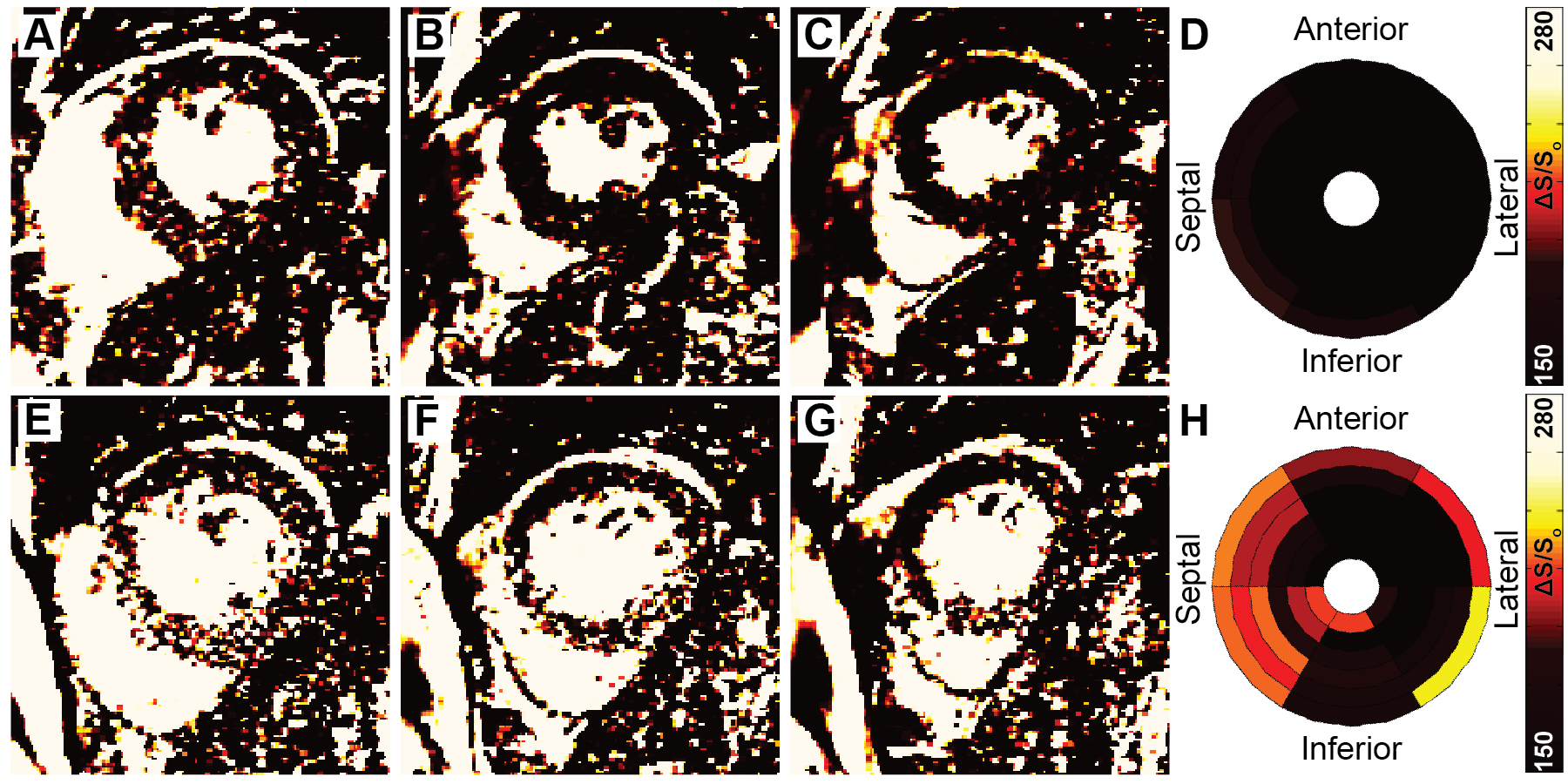


Figure S4. Representative patient with a large increase in diffuse fibrosis.

Representative ΔS/S_o_ maps at the (A) base, (B) mid ventricle, and (C) apex of a patient on hemodialysis for ESRD. (D) The bullseye plot revealed low ΔS/S_o_ values across the entire ventricle in this patient at baseline. At the follow up visit, ΔS/S_o_ maps demonstrated widespread elevations in the (E) base and regionally constrained elevations in the septal regions of the (F) mid ventricle and (G) apex of the left ventricle. (H) Diffuse elevation was evident across many slices of the LV, indicating that this patient had developed significant amounts of fibrosis over the follow up period. Divergence increased by 17.8 AU in this patient between visits.


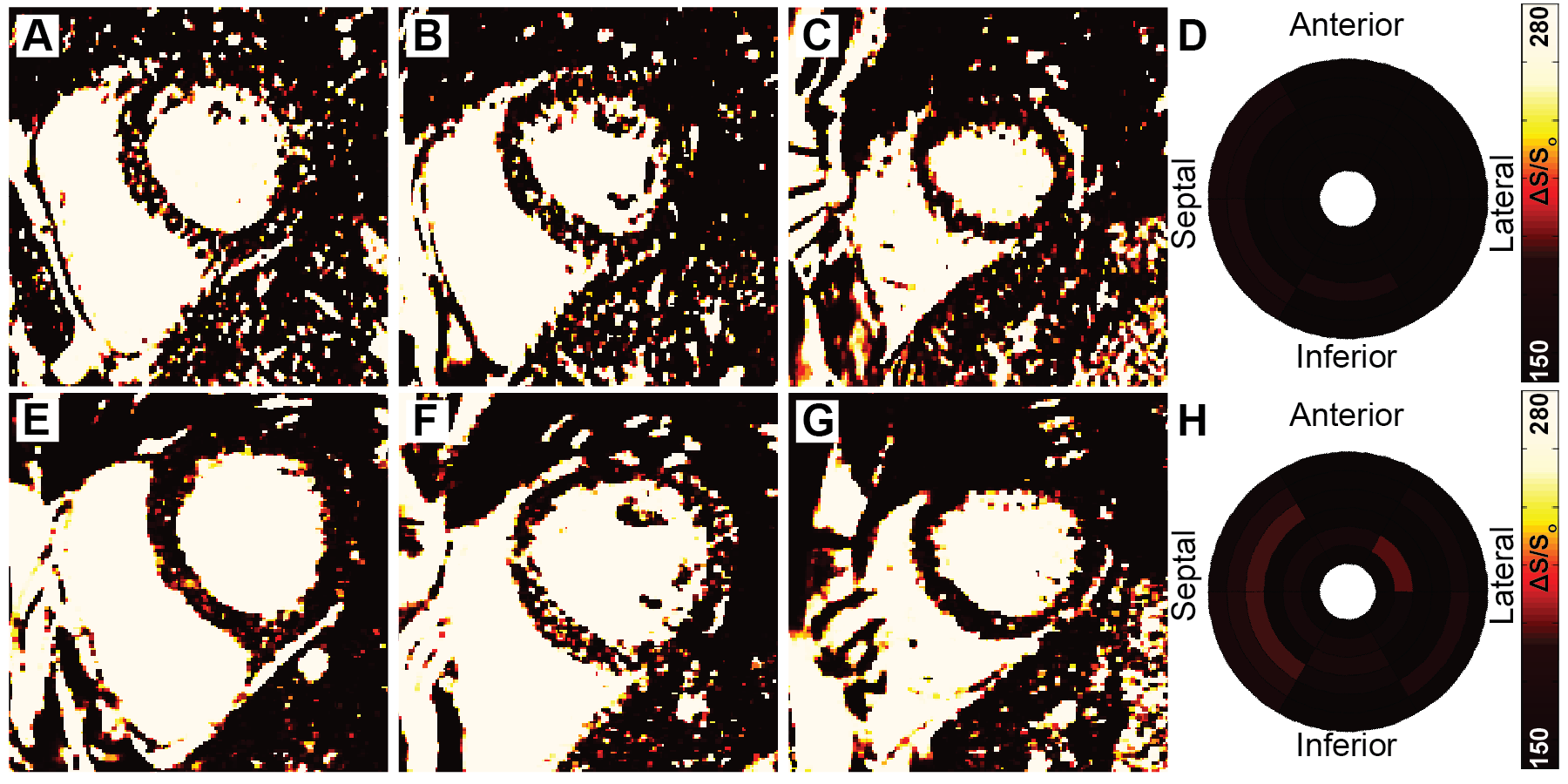


Figure S5. Minimal change in fibrosis over time.

At baseline, ΔS/S_o_ maps from this representative patient revealed minor scattered enhancement patterns in (A) basal, (B) mid ventricular, and (C) apical slices. (D) Average ΔS/S_o_ values remained low and within the normal healthy range across the entire ventricle in this patient. At follow up, myocardial ΔS/S_o_ remained low in the (E) base and (G) apex, with some increased values developing in the (F) mid ventricle, primarily in the septum. (H) The corresponding bullseye plot demonstrated patterns of minor elevations in this patient. Across the 2 study visits, global divergence increased by 3.9 AU.

Supplemental Table 2. Cardiac structure and function of 1 Year Follow-Up Subgroup

| **Variable** | **Baseline** | **Follow-Up** | **p** |
| --- | --- | --- | --- |
| Heart Rate (bpm) | 73.4 ± 10.1 | 71.7 ± 9.3 | 0.599 |
| BMI (kg/m^2^) | 29.5 ± 4.8 | 29.5 ± 4.3 | 0.901 |
| QRS Duration (ms) | 88.7 ± 15.1 | 93.8 ± 13.5 | 0.129 |
| QTc Interval (ms) | 463.2 ± 39.0 | 454.2 ± 28.9 | 0.709 |
| EDV (mL) | 121.5 ± 37.3 | 111.9 ± 39.7 | 0.062 |
| ESV (mL) | 46.5 ± 22.4 | 42.2 ± 21.0 | 0.213 |
| EF (mL) | 62.7 ± 7.7 | 62.9 ± 9.8 | 0.925 |
| Cardiac Output (L/min) | 5.4 ± 1.4 | 4.9 ± 1.6 | 0.248 |
| LV Mass (g) | 155.8 ± 46.1 | 163.2 ± 48.9 | 0.349 |
| LV Mass to Volume Ratio (g/ml) | 1.4 ± 0.5 | 1.4 ± 0.5 | 0.670 |
| LVMI (g/m^2^) | 80.3 ± 20.9 | 83.5 ± 20.8 | 0.481 |
| Septal Thickness (cm) | 1.2 ± 0.3 | 1.2 ± 0.2 | 0.547 |
| H/R Ratio | 0.5 ± 0.1 | 0.5 ± 0.1 | 0.970 |
| Data are presented as mean ± standard deviation. BMI: body mass index, QTc: corrected QT, EDV: end diastolic volume, ESV: end systolic volume, EF: ejection fraction, LV: left ventricle, LVMI: left ventricular mass index (indexed to BSA), H/R ratio: septal thickness/chamber radius. *p<0.05 was considered significant. | | | |

**Supplemental Table 3. Longitudinal changes in cardiac mechanics**

| **Variable** | **Baseline** | **Follow-Up** | ***p*** |
| --- | --- | --- | --- |
| Circumferential Shortening (%) | -29.9 ± 3.8 | -31.4 ± 5.1 | 0.44 |
| Systolic Strain Rate (%/s) | -157.8 ± 26.4 | -160.1 ± 32.6 | 0.86 |
| Diastolic Strain Rate (%/s) | 169.6 ± 37.7 | 178.4 ± 44.7 | 0.63 |
| Global Longitudinal Strain (%) | -21.3 ± 2.7 | -21.3 ± 4.0 | 0.98 |
| Longitudinal Systolic Strain Rate (%/s) | -107.4 ± 15.2 | -102.0 ± 23.4 | 0.53 |
| Longitudinal Diastolic Strain Rate (%/s) | 121.2 ± 24.4 | 101.0 ± 32.1 | 0.11 |
| Data are presented as mean ± standard deviation. Significance was determined using an analysis of variance test and p<0.05 was considered significant. | | | |

**
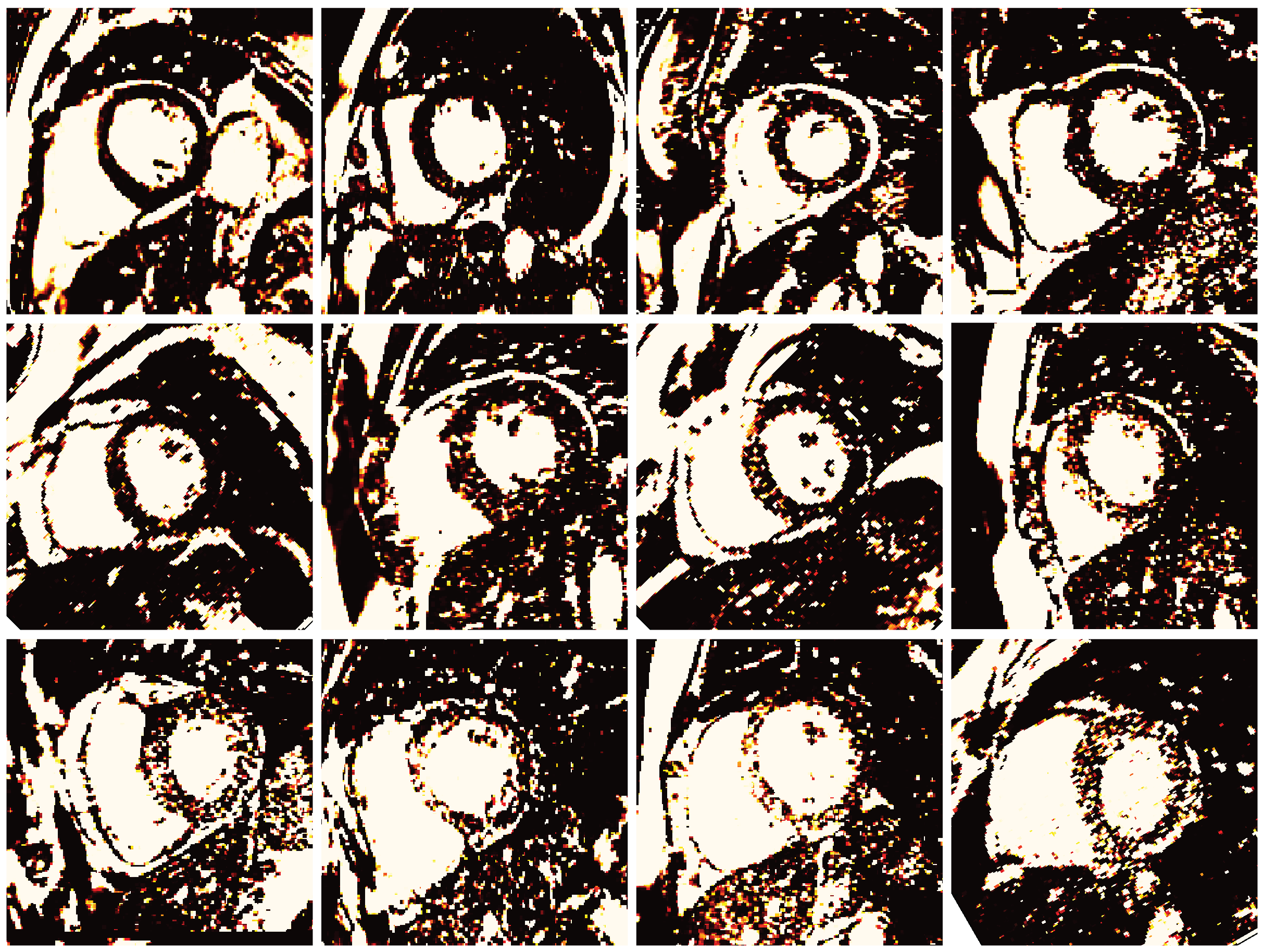
**

**Figure S6. Representative examples of individuals with ESRD and comorbid ventricular hypertrophy.** Mid-ventricular ΔS/S_o_ maps from twelve ESRD patients with elevated left ventricular mass index reveal a wide range of the magnitude and spatial distribution of ΔS/S_o_ elevation. ΔS/S_o_ values are uniformly low throughout the left ventricular myocardium of the patient shown in the top left image, similar to what is seen in control individuals (Figure S2). In subsequent images, progressively larger clusters of voxels are characterized by elevated ΔS/S_o_ values. In some patients (bottom row, first three panels) areas of dense focal enhancement can be seen in the septum. In others (final panel), diffuse elevations are observed throughout the left ventricular myocardium.

**
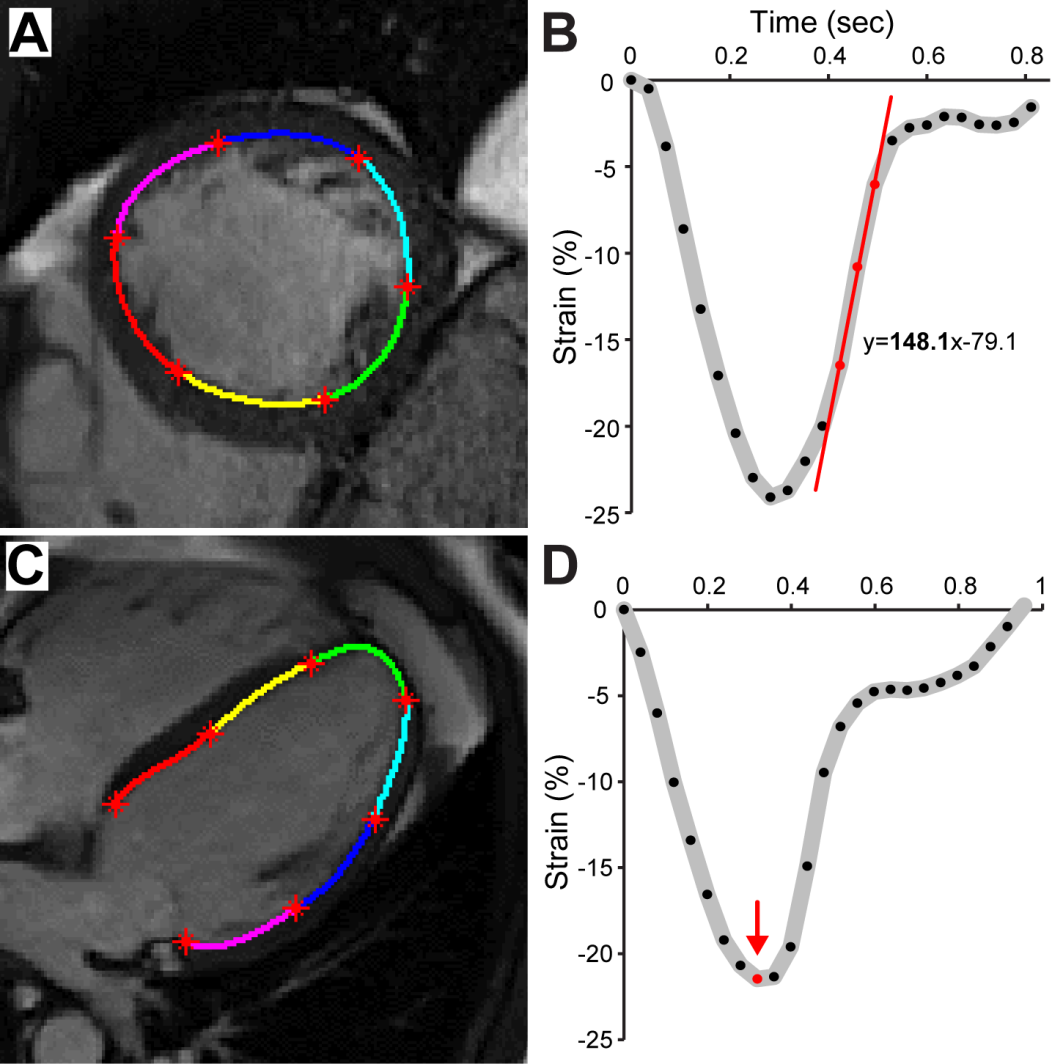
**

**Figure S7.** Analysis of cardiac mechanics using feature tracking as demonstrated in a healthy control.

**(A)** In each transverse slice, endocardial borders were manually defined at end diastole and automatically divided into 6 sectors. Sectors were followed through the cardiac cycle using feature tracking and manually corrected for errors in border definition. **(B)** Average circumferential shortening across all sectors was plotted as a function of the cardiac cycle (gray lines), and diastolic strain rate was calculated as the maximum slope during relaxation (red line, bold text). **(C)** Similarly, longitudinal strain as a function of cardiac phase was calculated from a 4-chamber longitudinal cine image. **(D)** The peak global longitudinal strain was defined by the minimum strain value (arrow).

### Blood Biomarkers and Correlations with LV Remodeling

Blood samples were collected by venipuncture at the time of imaging, allowed to clot 30 minutes, centrifuged (1300g, 10 minutes, 4°C) and stored (-80°C). Serum concentrations of TnT were analyzed by quantitative electrochemiluminescent immunoassay by ARUP Laboratories (0098803, Salt Lake City, UT, USA). MMP2 and MMP9 were measured by enzyme-linked immunosorbent assay (ELH-MMP2, ELH-MMP9 ELISA; Ray Biotech, Norcross, GA, USA). FGF23, PTH, TIMP1, and TIMP2 were analyzed by Milliplex® magnetic bead panels (HBNMAG-51K, HTMP2MAG-54K; EMD Millipore, Billerica, MA). Assays were duplicated and averaged for analysis.

Biomarkers of cardiac ischemia and remodeling—TnT and FGF23—were significantly elevated in patients with ESRD (Table S4). Increased PTH was evident in the ESRD cohort, as were markers of extracellular matrix turnover, TIMP1 and TIMP2. Correlations of divergence with TnT, PTH, TIMP1, and TIMP2 were statistically significant (Table S4). TnT, FGF23, MMP2, and PTH all correlated more strongly with LVMI (Table S4). In other studies, elevated blood levels of troponin T (TnT), fibroblast growth factor 23 (FGF23), and parathyroid hormone (PTH), which all correlate well with hypertrophy^1,2^, have been used as biomarkers of fibrosis. Additionally, galectin 3^3–5^ and matrix metalloproteinases (MMP) and tissue inhibitors of MMP (TIMP) are under investigation as fibrosis-specific markers in cardio-renal syndrome^6,7^. However, while changes in ventricular structure and function or elevated levels of specific blood biomarkers may be common in ESRD patients, they may not directly correlate with the development of cardiac fibrosis as a specific disease process^8^. Examination of cardiac fibrosis in ESRD patients is further complicated by a pattern of development that is intrinsically diffuse both in terms of magnitude and spatial distribution^9^. The absence of suitable techniques for non-invasive identification and quantification of cardiac fibrosis therefore remains an obstacle to identifying potential plasma biomarkers and developing new therapies in ESRD.

**Supplemental Table 4. Blood Biomarker Results.**

|  | **Control** | **ESRD** | ***p*** |  | **Spearman r (*p*)** | |
| --- | --- | --- | --- | --- | --- | --- |
| **Biomarker** | (n=25) | (n=16) |  |  | ***LVMI*** | ***Divergence*** |
| TnT (ng/ml) | undetected | 0.1  [0.0, 0.2] | <0.01 |  | 0.6 (<0.001) | 0.3 (0.043) |
| FGF23 (pg/ml) | 0.0  [0.0, 0.0] | 521.2  [29.5, 1210.9] | 0.001 |  | 0.3 (.030) | 0.3 (0.105) |
| PTH (pg/ml) | 139.7  [78.5, 167.6] | 544.2  [340.78, 901.96] | <0.001 |  | 0.4 (0.019) | 0.3 (0.035) |
| MMP2 (ng/ml) | 11.2  [6.9, 20.1] | 23.0  [11.9, 33.4] | 0.051 |  | 0.4 (0.014) | 0.3 (0.095) |
| MMP9 (ng/ml) | 931.9  [643.3, 1201.7] | 552.5  [347.9, 718.1] | 0.026 |  | -0.1 (0.136) | -0.2 (0.128) |
| TIMP1 (pg/ml) | 4336.0  [4090.5, 5757.5] | 6371.0  [4323.0, 7567.0] | 0.005 |  | 0.2 (0.201) | 0.4 (0.013) |
| TIMP2 (pg/ml) | 3255.0  [2555.5, 4408.5] | 4676.0  [2953.0, 5302.0] | 0.035 |  | 0.2 (0.278) | 0.4 (0.011) |
| LVMI: left ventricular mass index, TnT: troponin T, FGF: fibroblast growth factor, PTH: parathyroid hormone, MMP: matrix metalloproteinase, TIMP: tissue inhibitor of MMP. Group values presented as median [interquartile range]. p< 0.05 considered significant. | | | | | | |

Over the last decade, numerous studies in ESRD patients have revealed elevated circulating plasma levels of FGF23^1,10–12^, PTH^2,11^, and serum phosphates^11,13^ as potential factors driving ventricular hypertrophy. In particular, FGF23 levels are elevated earlier than PTH and serum phosphates^11^, correlate with left ventricular hypertrophy^1,14^, and are broadly linked to increased risk of cardiac mortality in patients with ESRD ^2,12,14^. Such findings have motivated development of new therapies including dietary intervention ^12,14,15^, oral phosphorous binders ^12,14^, and FGF23 blockade ^1,16,17^. In agreement with previous studies, our participants with ESRD demonstrated significantly elevated TnT, FGF23, and PTH. However, plasma FGF23 levels correlated more strongly with LV hypertrophy than with divergence. While prior investigations of MMPs and TIMPs in ESRD demonstrated variable plasma levels in patients with ESRD as a cohort^6,7^, we observed that elevated plasma TIMP1 and TIMP2 correlated with divergence but not hypertrophy. Aberrant TIMP signaling has been shown to increase cardiac fibroblast activation and collagen synthesis^12^ and is implicated in promoting fibrosis and contractile dysfunction in hypertrophy^18^ and heart failure^19^. When considering these findings, it is important to also consider the potential impact of ultrafiltration, residual excretion, and other factors that can influence blood serum levels unrelated to tissue fibrosis as described by Rasche et al^20^. Experimental therapies including torasemide^21^, spironolactone^22–24^, galectin 3 blockade ^4^, FGF23 blockade ^1,16,17^, and soluble guanylate cyclase activation^25^ each target molecular pathways believed to contribute to the development of hypertrophy and potentially fibrosis in the setting of ESRD. The ability to simultaneously and repeatedly quantify changes in ventricular structure, fibrosis, and function is crucial for the evaluation of such therapies, particularly those that are chronically administered. Cine balanced steady state free precession imaging is already a standard of care imaging sequence on most commercially available MRI scanners. The acquisition of image sets with high MT-weighting is already routinely performed as part of clinical examinations, and important parameters including ventricular structure and contractile function are already derived from such images. The addition of a second image set with low MT-weighting requires only a single parameter change, and enables the derivation of divergence data as presented in this study. Importantly, this approach simultaneously provides information about both the presence and extent of focal scar and global changes in total fibrotic burden. The added cost is one breath-hold for each imaging slice.

**References**

1. Faul, C. *et al.* FGF23 induces left ventricular hypertrophy. *J Clin Invest* **121,** 4393–4408 (2011).

2. Isakova, T. *et al.* Fibroblast growth factor 23 and risks of mortality and end-stage renal disease in patients with chronic kidney disease. *Jama* **305,** 2432–2439 (2011).

3. Ho, J. E. *et al.* Galectin-3, a Marker of Cardiac Fibrosis, Predicts Incident Heart Failure in the Community. *J. Am. Coll. Cardiol.* **60,** 1249–1256 (2012).

4. Martinez-Martinez, E. *et al.* Galectin-3 blockade inhibits cardiac inflammation and fibrosis in experimental hyperaldosteronism and hypertension. *Hypertension* **66,** 767–775 (2015).

5. Zamora, E. *et al.* Renal function largely influences Galectin-3 prognostic value in heart failure. *Int J Cardiol* **177,** 171–177 (2014).

6. Pawlak, K., Mysliwiec, M. & Pawlak, D. Peripheral blood level alterations of MMP-2 and MMP-9 in patients with chronic kidney disease on conservative treatment and on hemodialysis. *Clin Biochem* **44,** 838–843 (2011).

7. Rysz, J. *et al.* Serum metalloproteinases MMP-2, MMP-9 and metalloproteinase tissue inhibitors TIMP-1 and TIMP-2 in patients on hemodialysis. *Int Urol Nephrol* **43,** 491–498 (2011).

8. Yamada, S. *et al.* Mechanical Dyssynchrony Precedes QRS Widening in ATP‐Sensitive K+ Channel–Deficient Dilated Cardiomyopathy. *J. Am. Heart Assoc.* **2,** 2:e000410 (2013).

9. Saran, R. *et al.* US Renal Data System 2016 Annual Data Report: Epidemiology of Kidney Disease in the United States. *Am J Kidney Dis* **69,** A7-a8 (2017).

10. Isakova, T. *et al.* Effects of dietary phosphate restriction and phosphate binders on FGF23 levels in CKD. *Clin J Am Soc Nephrol* **8,** 1009–1018 (2013).

11. Isakova, T. *et al.* Fibroblast growth factor 23 is elevated before parathyroid hormone and phosphate in chronic kidney disease. *Kidney Int* **79,** 1370–1378 (2011).

12. Gutierrez, O. M. *et al.* Fibroblast growth factor 23 and mortality among patients undergoing hemodialysis. *N Engl J Med* **359,** 584–592 (2008).

13. Gutierrez, O. M. Increased serum phosphate and adverse clinical outcomes: unraveling mechanisms of disease. *Curr. Opin. Nephrol. Hypertens.* **20,** 224–228 (2011).

14. Gutierrez, O. M. *et al.* Fibroblast growth factor 23 and left ventricular hypertrophy in chronic kidney disease. *Circulation* **119,** 2545–2552 (2009).

15. Gutierrez, O. M. *et al.* Dietary patterns and risk of death and progression to ESRD in individuals with CKD: a cohort study. *Am J Kidney Dis* **64,** 204–213 (2014).

16. Di Marco, G. S. *et al.* Treatment of established left ventricular hypertrophy with fibroblast growth factor receptor blockade in an animal model of CKD. *Nephrol Dial Transpl.* **29,** 2028–2035 (2014).

17. Hassan, M. *et al.* Interplay of parathyroid hormone and aldosterone antagonist in prevention of heart failure hospitalizations in chronic kidney disease. *J. Renin-Angiotensin-Aldosterone Syst.* **15,** 278–85 (2014).

18. Vanhoutte, D. & Heymans, S. TIMPs and cardiac remodeling: ‘Embracing the MMP-independent-side of the family’. *J Mol Cell Cardiol* **48,** 445–453 (2010).

19. Lombardi, R. *et al.* Myocardial collagen turnover in hypertrophic cardiomyopathy. *Circulation* **108,** 1455–1460 (2003).

20. Rasche, F. M. *et al.* Modern peptide biomarkers and echocardiography in cardiac healthy haemodialysis patients. *BMC Nephrol.* **18,** 175 (2017).

21. Ló pez, B., González, A., Hermida, N., Laviades, C. & Díez, J. Myocardial fibrosis in chronic kidney disease: potential benefits of torasemide. *Kidney Int.* **74,** S19–S23 (2008).

22. Jellis, C. *et al.* Association of Imaging Markers of Myocardial Fibrosis With Metabolic and Functional Disturbances in Early Diabetic Cardiomyopathy. *Circ. Cardiovasc. Imaging* **4,** 693–702 (2011).

23. Coelho-Filho, O. R. *et al.* Cardiac magnetic resonance assessment of interstitial myocardial fibrosis and cardiomyocyte hypertrophy in hypertensive mice treated with spironolactone. *J Am Hear. Assoc* **3,** e000790 (2014).

24. Pitt, B. *et al.* Spironolactone for heart failure with preserved ejection fraction. *N Engl J Med* **370,** 1383–1392 (2014).

25. Dubin, R. F. & Shah, S. J. Soluble Guanylate Cyclase Stimulators: a Novel Treatment Option for Heart Failure Associated with Cardiorenal Syndromes? *Curr Hear. Fail Rep* **13,** 132–139 (2016).
